# Osteoporosis and Stress Urinary Incontinence in Women: A National Health Insurance Database Study

**DOI:** 10.3390/ijerph17124449

**Published:** 2020-06-21

**Authors:** Ming-Cheng Wei, Ying-Hsiang Chou, Yi-Sun Yang, Edy Kornelius, Yu-Hsun Wang, Chien-Ning Huang

**Affiliations:** 1 Institute of Medicine, Chung Shan Medical University, Taichung 40201, Taiwan; fazen.tw@yahoo.com.tw; 2 Department of Neurosurgery, Lee General Hospital, Yuanli Town, Miaoli 35845, Taiwan; 3 Department of Radiation Oncology, Chung Shan Medical University Hospital, Taichung 40201, Taiwan; hideka@gmail.com; 4 Department of Medical Imaging and Radiological Sciences, Chung Shan Medical University Hospital, Taichung 40201, Taiwan; 5 Division of Endocrinology and Metabolism, Department of Internal Medicine, Chung Shan Medical University Hospital, Taichung 40201, Taiwan; monica119@gmail.com (Y.-S.Y.); kornelius82@gmail.com (E.K.); 6 Department of Medical Research, Chung Shan Medical University Hospital, Taichung 40201, Taiwan; cshe731@csh.org.tw

**Keywords:** urinary incontinence, osteoporosis, women, retrospective study

## Abstract

We aimed to determine the influence of osteoporosis and stress urinary incontinence in women. We hypothesized that women with osteoporosis had an increased risk of stress urinary incontinence. This retrospective study used data from the Taiwan Longitudinal Health Insurance database from 2005–2009. The study population was screened to identify women (age ≥ 40 years) newly diagnosed with osteoporosis (ICD-9-CM code = 733.0, 733.1). The osteoporosis cohort included 6125, and the non-osteoporosis cohort included 12,250 participants. The newly diagnosed stress urinary incontinence incidence was calculated to determine the influence of osteoporosis and stress urinary incontinence. We used the Cox proportional hazards model to predict the effects of stress urinary incontinence and the Kaplan–Meier analysis to estimate the cumulative incidence of stress urinary incontinence in women. Participants with osteoporosis experienced a 1.79 times higher risk than that of the non-osteoporosis group (95% CI = 1.28–2.51) for stress urinary incontinence, regardless of age. We did not observe a higher risk of stress urinary incontinence in participants with pathological fractures compared to those with simple osteoporosis. Our data emphasized that physicians and nurses should conduct urinary incontinence screening in women with osteoporosis to recommend proper treatment, medical help or to bring the disorder to light.

## 1. Introduction

Osteoporosis (OS) is defined by the National Institutes of Health (NIH) as a high risk of fractures caused by changes in bone strength [1]. OS can cause pain, hunchback appearance, and reduced mobility, thereby causing muscle dysfunction and atrophy. Urinary incontinence (UI) is defined by the International Continence Society as any involuntary urinary leakage. UI includes two major types: stress and urgency. Stress UI (SUI) is associated with reduced physical activity, increased risk of falls, and is the second leading cause of long-term care admission [2]. Although SUI is not directly related to aging, its prevalence and severity increase with age [3].

Initially, we set out to compare pelvic floor disorders, but there were not enough cases of fecal incontinence or pelvic organ prolapse. This comparison can be found in Supplementary Table S1. OS is caused by the gradual aging of bone metabolism, whereas SUI is caused by the loosening of the soft tissue and muscle ligaments in the pelvic floor. SUI is one of the major pelvic floor diseases. It is more common than fecal incontinence and pelvic organ prolapse.

OS is associated with sarcopenia, an age-related muscular disease that affects muscle size and function and results in further loss of connective tissue tension [4]. Thus, the abovementioned pelvic floor diseases may be related to sarcopenia. Muscle weakness may weaken the supporting strength of the pelvic floor, thereby causing these diseases, especially SUI [5,6,7]. These debilitating processes are ameliorated by the regulation of estrogen and selective estrogen receptor modulators [8,9].

A previous study conducted from February 2011 to March 2012, which included 78 postmenopausal and 30 premenopausal women with SUI and 57 continent postmenopausal and 20 premenopausal unaffected women, revealed that osteoporosis was more prevalent in women with SUI (*p* < 0.05). Estradiol levels were decreased in the postmenopausal and premenopausal women with SUI compared to those in the control group. Women with low estradiol levels usually had a T score of ≤−2.5 and had osteoporosis [10].

Prospective studies revealed that SUI in women who developed osteoporosis may have been caused by low estradiol levels; however, they failed to reveal the association between OS and SUI. Hence, we used the Taiwan National Health Insurance Research Database Longitudinal Health Insurance Database to determine the influence of OS and SUI. We hypothesized that women with osteoporosis had an increased risk of UI.

## 2. Materials and Methods

This study was a retrospective cohort study that analyzed the Longitudinal Health Insurance Database from Taiwan, which served as a dataset for our cohort, and provided samples for 1 million participants of the 23 million insured individuals in the Taiwan National Health Insurance Research Database. The study was approved by the Institutional Review Board of the Affiliated Hospital of Chung Shan Medical University (Ethical approval number: CS13161). The requirement for obtaining informed consent from participants was waived because of the retrospective nature of the research.

**Figure 1:**
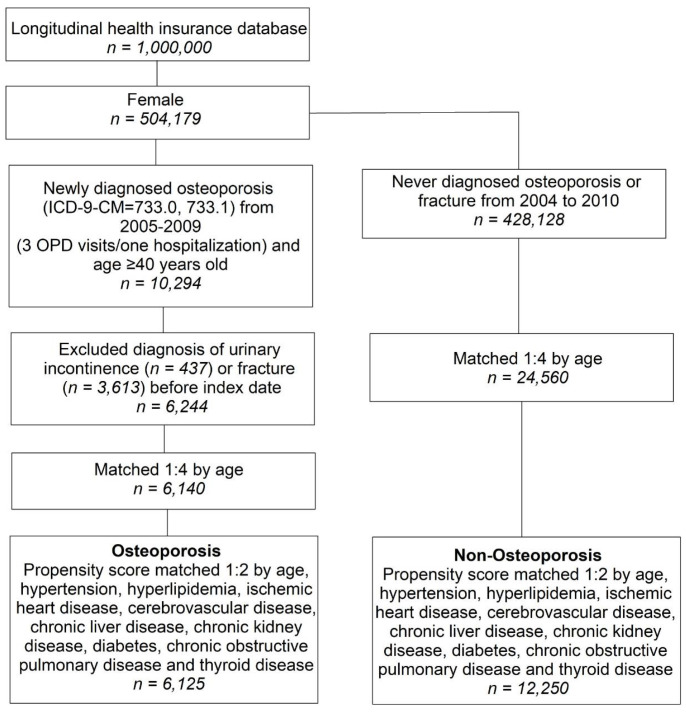
Participant selection process for the OS and Non-OS groups. OS, osteoporosis; OPD, Outpatient Department.

The selection process for the participants in the two study groups, the osteoporosis (OS) group and the non-osteoporosis (Non-OS) group, is depicted in Figure 1. The study population in the 2005–2009 database was screened for newly diagnosed OS women aged 40 years or older (ICD-9-CM code = 733.0, 733.1). The index date for this cohort was defined as the date when the participants were first diagnosed with OS. We further excluded participants who were diagnosed with UI (ICD-9-CM = 625.6) or fracture (ICD-9-CM = 800–829) before the index date. The Non-OS group included participants who were not diagnosed with OS between 2004 and 2010.

Outcome measurements were defined as the diagnosis of SUI (ICD-9-CM = 625.6). To ensure the accuracy of the diagnosis, we included participants with at least three outpatient visits or who were admitted to the hospital at least once. Participants were followed-up until 31 December 2010, or until another end point occurred, including the diagnosis of SUI or patient withdrawal from the insurance plan.

Baseline characteristics were age, hypertension (ICD-9-CM code = 401–405), hyperlipidemia (ICD-9-CM code = 272.0–272.4), diabetes (ICD-9-CM code = 250), cerebrovascular disease (ICD-9-CM code = 430–438) ischemic heart disease (ICD-9-CM code = 410–414), thyroid disease (ICD-9-CM code = 240–246), chronic obstructive pulmonary disease (ICD-9-CM code = 491, 492, 496), chronic liver disease (ICD-9-CM code = 571), and chronic kidney disease (ICD-9-CM code = 585).

Treatments related to these possible comorbidities were clearly limited to at least three outpatient visits or at least one hospitalization within one year before the index date.

First, we performed 1:4 age matching to obtain an index date corresponding to the non-osteoporosis group. Then, based on baseline characteristics, we performed 1:2 propensity score matching to control for the heterogeneity between the OS and Non-OS groups.

The baseline characteristics of the two groups were compared using the independent *t*-test for continuous variables and the chi-squared test for categorical variables. Kaplan–Meier analysis was also performed to estimate the cumulative probability of SUI in both groups, and a log-rank test was used to test to determine statistical significance. The Cox proportional hazard model was then used to analyze the hazard ratios for OS or other variables. SPSS 18.0 (SPSS Inc., Chicago, IL, USA) software was used for statistical analyses. A *p*-value < 0.05 was considered statistically significant.

## 3. Results

Study participants who met the screening criteria included 6125 participants in the OS cohort and 12,250 participants in the Non-OS cohort after propensity score matching (Table 1). There were no statistically significant differences in age and comorbidity distribution between the groups after the propensity scores were matched.

To control the impact on SUI, this study further included the following potential comorbidities related to SUI. In the distribution analysis of comorbidities, including hypertension, hyperlipidemia, diabetes, cerebrovascular disease, thyroid disease, chronic obstructive pulmonary disease, ischemic heart disease, chronic liver disease, and chronic kidney disease, the proportion of participants with osteoporosis was not higher than that of participants without osteoporosis.

**Figure 2:**
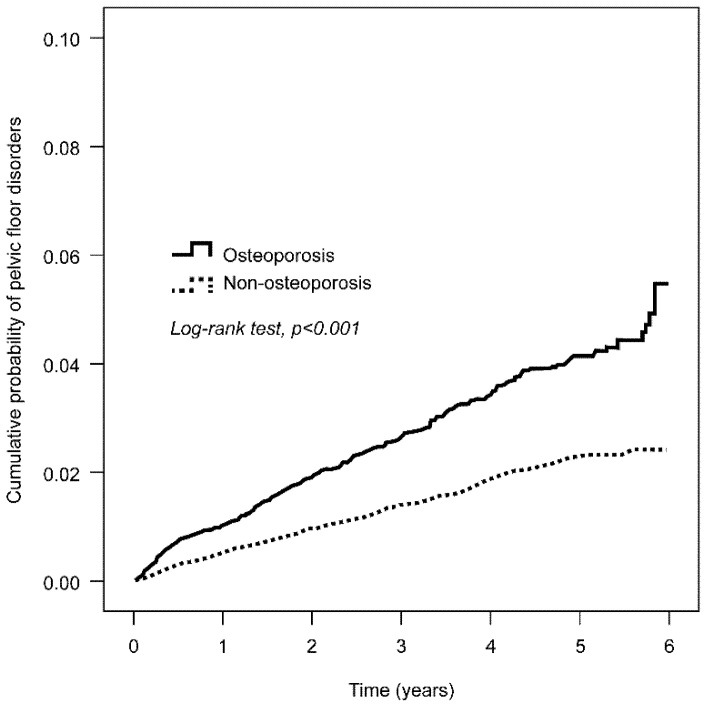
Kaplan–Meier analysis of the cumulative probability of pelvic floor disorders at the 6-year follow-up. At the 6-year follow-up, the cumulative incidence for developing urinary incontinence in the OS group (black line) is higher than in the Non-OS group (dotted line). The *p*-value is less than 0.001 after the log-rank test.

Kaplan–Meier analysis indicated that at the end of the 6-year follow-up period, the cumulative incidence of UI was higher in the OS than in the Non-OS group (*p* = 0.001 after the log-rank test) (Figure 2). Table 2 shows that the adjusted hazard ratio for the OS was 1.79 times that of the Non-OS group (95% CI = 1.28–2.51). Considering other comorbidities, we analyzed the relationship between OS and UI by stratification and found that only hyperlipidemia reached statistical significance (1.70 times [95% CI = 1.11–2.58]).

Table 3 shows that the risk of UI was significantly higher in the OS than in the Non-OS group, regardless of age stratification. When adjustments were made for age, hypertension, hyperlipidemia, ischemic heart disease, cerebrovascular disease, chronic liver disease, chronic kidney disease, diabetes, chronic obstructive pulmonary disease, and thyroid disease, the risk of SUI was significantly increased in participants with OS compared to those without OS. In participants aged 55 to 69, those with OS had a higher risk of SUI than those without OS (adjusted HR of 2.74 (95% CI = 1.62–4.64)).

In Table 4, we analyze the hazard ratios for OS compared the outcomes of SUI. The outcomes were simple OS and pathological fractures. The results show that pathological fractures were not associated with a significantly higher risk of UI than simple OS.

## 4. Discussion

This retrospective cohort study investigated the relationship between OS and SUI in women. We found that the risk of SUI was 1.79 times higher in women with OS than in those without OS, regardless of age. The risk of SUI was also higher in participants with simple OS than in those with pathological fractures, but the difference was not statistically significant.

In the cross-sectional analysis of the 2005–2006 US National Health and Nutrition Examination Survey, the weighted prevalence of at least one pelvic floor symptom was 24% [11]. The prevalence of UI in older women ranged from 21–28% [12,13,14,15]. An early study of women with OS reported even higher rates, and nearly 40% of women (163/412) reported more than one leak per week. Moreover, the prevalence of urine retention was high and was accompanied by urgency [12]. UI with urgency was also an independent risk factor for falls and low-velocity trauma fractures in older women. According to statistics, UI affects one third of the female population; however, less than 20% of participants actually seek medical treatment.

Similarly, the prevalence of UI is also high among women with OS. Because UI limits physical activity, reduces muscle mass and function, and increases the risk of falls [16] and fractures, screening for UI should be a regular part of OS treatment.

The mechanism by which SUI increases in women with OS is unclear. On the basis of our findings, we suggest two possible hypotheses. The first is that there is a common pathophysiology between pelvic floor disorders and OS. The presence of bone mineral density reduction, OS, and fractures reflects defects in bone connective tissue, including mass, matrix, and microstructural abnormalities [17,18]. The second hypothesis is that the decline in height caused by aging is mainly due to vertebral compression caused by OS [19]. OS is more common in women, and older women show a faster decline in height than older men [20]. A decrease in height causes an increase in intra-abdominal pressure and associated symptoms, such as epigastralgia, UI, and hemorrhoids [21]. Changes in spinal curvature and compression fractures of the spine result in an increase in intra-abdominal pressure, by pushing on the pelvic floor muscles. Posterior kyphosis and the subsequent elevation in intra-abdominal pressure may explain the relationship between vertebral fractures and esophageal hiatal hernia, which are detected early in Japanese women [22]. A study revealed that factors associated with decreased height were similar to those associated with OS; for example, increased body weight and estrogen therapy could prevent height loss [23].

In this study, the adjusted hazard ratio for the OS group was 1.79 times higher than that of the Non-OS group. In one prospective cohort study of 12,570 female participants seen by a general physician, OS was associated with self-reported overactive bladder symptoms but not with UI [24]. In another population-based interview study that included 3010 men and women, OS emerged as a significant correlate with self-reported pelvic floor dysfunction (OR = 1.8, *p* = 0.49), defined as current or past UI, FI, POP, bladder or vaginal repair [5].

Among the effects of comorbidities, we found that participants with hyperlipidemia were 1.70 times more likely to have SUI. This may reflect a similar pathophysiological process in bone and other collagen-based supporting connective tissues.

The main limitation of this study was its retrospective design. Because the health insurance data include claims data, there are no data sources such as inspection data, life history questionnaires, height and weight, among others. This study does not provide further insight into the major mechanisms of progression and exacerbation of SUI from OS. However, the main strength of the study was that it was based on a large national database and an unbiased assessment of the diagnosis by specialists.

## 5. Conclusions

An increased risk of SUI was demonstrated in participants with OS. Our findings emphasize the need for physicians and nurses to conduct SUI screening to recommend proper treatment, medical help or to bring the disorder to light for women with OS.

## Figures and Tables

**Table 1:** Demographic characteristics of participants with and without osteoporosis.

	Before Propensity Score Matching					After Propensity Score Matching				
	Osteoporosis (*N* = 6140)		Non-Osteoporosis (*N* = 24,560)			Osteoporosis (*N* = 6125)		Non-Osteoporosis (*N* = 12,250)		
	*n*	%	*n*	%	*p*-Value	*n*	%	*n*	%	*p*-Value
Age					1					0.560
40–54	1373	22.4	5492	22.4	-	1368	22.3	2653	21.7	-
55–69	2449	39.9	9796	39.9	-	2443	39.9	4907	40.1	-
≥70	2318	37.8	9272	37.8	-	2314	37.8	4690	38.3	-
Mean ± SD	65.4 ± 11.4		65.4 ± 11.4		1	65.4 ± 11.4		65.6 ± 11.3		0.212
Hypertension	2332	38.0	8384	34.1	<0.001 *	2325	38.0	4709	38.4	0.527
Hyperlipidemia	950	15.5	2727	11.1	<0.001 *	937	15.3	1884	15.4	0.885
Ischemic heart disease	724	11.8	2210	9.0	<0.001 *	719	11.7	1467	12.0	0.640
Cerebrovascular disease	438	7.1	1545	6.3	0.016 *	435	7.1	880	7.2	0.840
Chronic liver disease	343	5.59	930	3.79	<0.001 *	333	5.44	671	5.48	0.909
Chronic kidney disease	64	1.0	362	1.5	0.010 *	64	1.0	108	0.9	0.279
Diabetes	929	15.1	3692	15.0	0.848	928	15.2	1877	15.3	0.761
COPD	263	4.3	720	2.9	<0.001 *	260	4.2	516	4.2	0.917
Thyroid disease	237	3.9	467	1.9	<0.001 *	222	3.6	415	3.4	0.408
Type										
Osteoporosis	5565	90.6	-	-	-	5550	90.6	-	-	-
Pathologic fracture	575	9.4	-	-	-	575	9.4	-	-	-

COPD: chronic obstructive pulmonary disease; * (*p* < 0.05); independent *t*-test was used for continuous variables and the chi-squared test for categorical variables; no significant differences in age and comorbidity distribution were found between the two groups.

**Table 2:** Cox proportional hazard model of osteoporosis and risk of urinary incontinence through comorbidity stratification.

Characteristics	Participants with Stress Urinary Incontinence	Observed Person-Years	Incidence Density (per 1000 Person-Years)	Crude HR	95% CI	Adjusted HR ^a^	95% CI
Osteoporosis							
No	71	46,569	1.5	1	-	1	-
Yes	65	23,787	2.7	1.80 *	1.28–2.51 *	1.79 *	1.28–2.51 *
Age							
40–54	30	16,258	1.8	1	-	1	-
55–69	57	28,991	2.0	1.07	0.68–1.66	0.96	0.61–1.51
≥70	49	25,107	2.0	1.05	0.67–1.66	0.89	0.54–1.45
Hypertension	62	26,237	2.4	1.41 *	1.003–1.97 *	1.34	0.92–1.96
Hyperlipidemia	32	10,608	3.0	1.73 *	1.16–2.57 *	1.70 *	1.11–2.58 *
Ischemic heart disease	21	8127	2.6	1.40	0.88–2.23	1.22	0.75–1.99
Cerebrovascular disease	7	4625	1.5	0.77	0.36–1.64	0.66	0.30–1.43
Chronic liver disease	10	3711	2.7	1.42	0.75–2.71	1.36	0.71–2.61
Diabetes	19	10,193	1.9	0.95	0.59–1.55	0.73	0.44–1.21
COPD	9	2680	3.4	1.78	0.91–3.50	1.75	0.88–3.48
Thyroid disease	7	2406	2.9	1.53	0.72–3.27	1.54	0.72–3.30

CI, confidence interval; COPD, chronic obstructive pulmonary disease; HR, hazard ratio; * (*p* < 0.05); the Cox proportional hazard model was used to analyze the hazard ratios for osteoporosis or other variables. ^a^ Adjusted for age, hypertension, hyperlipidemia, ischemic heart disease, cerebrovascular disease, chronic liver disease, chronic liver disease, chronic kidney disease, diabetes, chronic obstructive pulmonary disease, and thyroid disease.

**Table 3:** Subgroup analysis of the osteoporosis and non-osteoporosis groups in terms of the presence of urinary incontinence using the Cox proportional hazard model.

	Osteoporosis		Non-Osteoporosis			
Age ^a^	*N*	Participants with stress urinary incontinence	*N*	Participants with stress urinary incontinence	HR	95% CI
40–54	1368	14	2653	16	1.67	0.82–3.44
55–69	**2443**	**33**	**4907**	**24**	2.74 *	1.62–4.64 *
≥70	2314	18	4690	31	1.13	0.63–2.02

CI, confidence interval; HR, hazard ratio; * (*p* < 0.05); the Cox proportional hazard model was used to analyze the hazard ratios for osteoporosis or other variables; ^a^ adjusted for age, hypertension, hyperlipidemia, ischemic heart disease, cerebrovascular disease, chronic liver disease, diabetes, chronic obstructive pulmonary disease, and thyroid disease.

**Table 4:** Cox proportional hazard model of participants with stress urinary incontinence.

	No. of Events	Observed Person-Years	Incidence Density (per 1000 Person-Years)	Crude HR	95% CI	Adjusted HR	95% CI
Type ^a^							
No	71	46,569	1.5	1	-	1	-
Osteoporosis	60	22,000	2.7	1.79 *	1.27–2.53 *	1.79 *	1.27–2.52 *
Pathologic fracture	5	1787	2.8	1.82	0.73–4.5	1.83	0.73–4.58

^a^ Adjusted for age, hypertension, hyperlipidemia, ischemic heart disease, cerebrovascular disease, chronic liver disease, chronic kidney disease, diabetes, chronic obstructive pulmonary disease, and thyroid disease. * The Cox proportional hazard model was used to analyze the hazard ratios for osteoporosis or other variables. Osteoporosis is classified as simple osteoporosis or pathological fractures.

## Supplementary Materials

The following are available online at https://www.mdpi.com/1660-4601/17/12/4449/s1, Table S1: Cox proportional hazard model.
