# Supplementary material for: Osteoporosis and Stress Urinary Incontinence in Women: A National Health Insurance Database Study
Source: Int J Environ Res Public Health. 2020 Jun 21;17(12):4449. doi: 10.3390/ijerph17124449 (PMC7344808; doi:10.3390/ijerph17124449)
Supplement: Supplementary file 1 [file ijerph-17-04449-s001.pdf]

International Journal of
Environmental Research and Public Health

MDPI

Supplementary Table S1. Cox proportional hazard model.

	No. of event	Observed person-years	Incidence density (per 1000 person-years)	Crude HR	95% CI	Adjusted HR	95% CI
Model 1† (outcome: **pelvic floor disorders**)							
Type							
No	261	57892	4.5	1		1	
Osteoporosis	220	26552	8.3	**1.84**	**1.54-2.21**	**1.87**	**1.56-2.24**
Pathologic fracture	31	2540	12.2	**2.65**	**1.83-3.85**	**2.24**	**1.53-3.26**
Model 2† (outcome: **pelvic organ prolapse**)							
Osteoporosis							
No	25	58452	0.4	1		1	
Yes	13	29693	0.4	1.02	0.52-2.00	1.03	0.53-2.01
Model 3‡ (outcome: **fecal incontinence**)							
Osteoporosis							
No	3	58506	0.1	1		1	
Yes	2	29715	0.1	1.31	0.22-7.84	1.24	0.21-7.48
Model 4† (outcome: **urinary incontinence**)							
Type							
No	233	57952	4.0	1		1	
Osteoporosis	207	26581	7.8	**1.94**	**1.61-2.34**	**1.98**	**1.64-2.38**
Pathological fracture	29	2541	11.4	**2.78**	**1.89-4.09**	**2.30**	**1.56-3.40**

†Adjusted for age, sex, hypertension, hyperlipidemia, ischemic heart disease, cerebrovascular disease, chronic liver disease, chronic kidney disease, diabetes, chronic obstructive pulmonary disease, and thyroid disease. ‡Adjusted for age, sex, hypertension, hyperlipidemia, ischemic heart disease, and cerebrovascular disease. OS is classified as simple OS or pathological fractures. The outcomes of the three pelvic floor diseases, including urinary incontinence, fecal incontinence, and pelvic organ prolapse, were compared.
